# Crystal structure and Hirshfeld surface analysis of dimethyl (3a*S*,6*R*,6a*S*,7*S*)-2-(2,2,2-tri­fluoro­acet­yl)-2,3-di­hydro-1*H*,6*H*,7*H*-3a,6:7,9a-di­epoxy­benzo[*de*]iso­quinoline-3a^1^,6a-di­carboxyl­ate

**DOI:** 10.1107/S2056989018014305

**Published:** 2018-10-19

**Authors:** Zeliha Atioğlu, Mehmet Akkurt, Flavien A. A. Toze, Pavel V. Dorovatovskii, Narmina A. Guliyeva, Humay M. Panahova

**Affiliations:** a İlke Education and Health Foundation, Cappadocia University, Cappadocia Vocational College, The Medical Imaging Techniques Program, 50420 Mustafapaşa, Ürgüp, Nevşehir, Turkey; b Department of Physics, Faculty of Sciences, Erciyes University, 38039 Kayseri, Turkey; c Department of Chemistry, Faculty of Sciences, University of Douala, PO Box 24157, Douala, Republic of Cameroon; d National Research Center "Kurchatov Institute", Moscow, Russian Federation; e Organic Chemistry Department, Baku State University, Z. Xalilov Str. 23, Az 1148, Baku, Azerbaijan; f State Economic University of Azerbaijan, Istiqlaliyyat st., 6., AZ1001, Baku, Azerbaijan

**Keywords:** crystal structure, di­hydro­furan ring, tetra­hydro­furan ring, fused hexa­cyclic system, piperidine ring, Hiershfeld surface analysis

## Abstract

The title mol­ecule, C_18_H_16_F_3_NO_7_, comprises a fused cyclic system containing four five-membered (two di­hydro­furan and two tetra­hydro­furan) rings and one six-membered (piperidine) ring. The five-membered di­hydro­furan and tetra­hydro­furan rings adopt envelope conformations, and the six-membered piperidine ring adopts a distorted chair conformation. Intra­molecular O**⋯**F inter­actions help to stabilize the conformational arrangement. In the crystal structure, mol­ecules are linked by weak C—H⋯O and C—H⋯F hydrogen bonds, forming a three-dimensional network. The Hirshfeld surface analysis confirms the dominant role of H⋯H contacts in establishing the packing.

## Abstract 2

In the mol­ecular structure of the title compound, two di­hydro­furan and two tetra­hydro­furan rings as well as one piperidine ring are fused together. In the crystal, mol­ecules are linked by C—H⋯O and C—H⋯F hydrogen bonds, forming a three-dimensional network.

## Chemical context   

Non-covalent inter­actions, such as hydrogen, aerogen, halogen, chalcogen, pnicogen, tetrel and icosa­gen bonds, as well as *n*–π*, π–π stacking, π–cation, π–anion and hydro­phobic inter­actions, have an impact on the synthesis, catalysis and design of materials and on biological processes (Shikhaliyev *et al.*, 2018 ▸; Hazra *et al.*, 2018 ▸). These weak forces can also control or organize the aggregation, conformation, tertiary and quaternary structure of a mol­ecule, and its stabilization or other particular properties (Legon, 2017 ▸; Mahmudov *et al.*, 2017*a* ▸,*b* ▸). In comparison with well-established hydrogen and halogen bonds (Cavallo *et al.*, 2016 ▸; Mahmoudi *et al.*, 2018 ▸; Vandyshev *et al.*, 2017 ▸), chalcogen, pnicogen, tetrel and icosa­gen bonds are much less explored (Mahmudov *et al.*, 2017*a* ▸; Scheiner, 2013 ▸; Mikherdov *et al.*, 2016 ▸).

The title compound, C_18_H_16_F_3_NO_7_, has a 7-oxabi­cyclo[2.2.1]heptene scaffold, thus making it a potential tool for the design and synthesis of new organic materials with various useful properties such as electronic materials, molecular tweezers, *etc* (Borisova *et al.*, 2018*a* ▸,*b* ▸). During the structure determination, we noted rather unusual intra­molecular O⋯F inter­actions. Here we report the synthesis, mol­ecular and crystal structure of this compound as well as a Hirshfeld surface analysis.
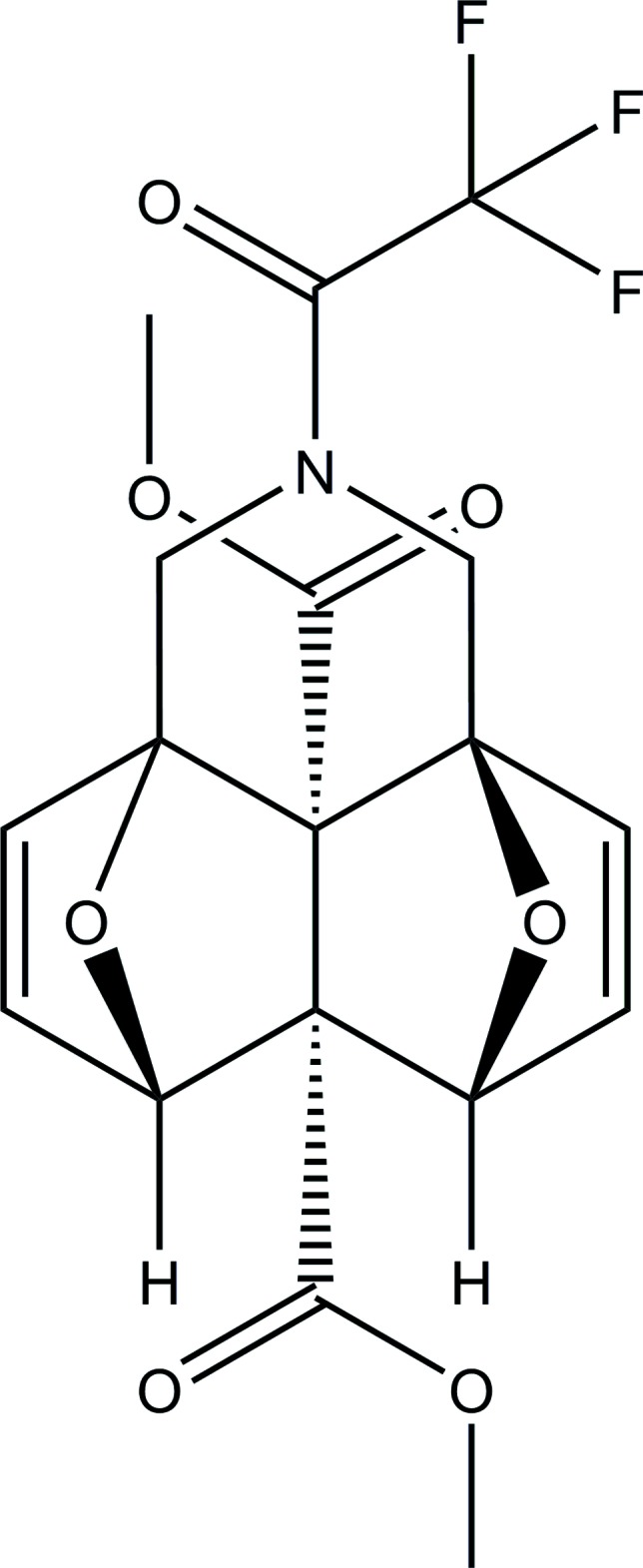


## Structural commentary   

**Figure 1:**
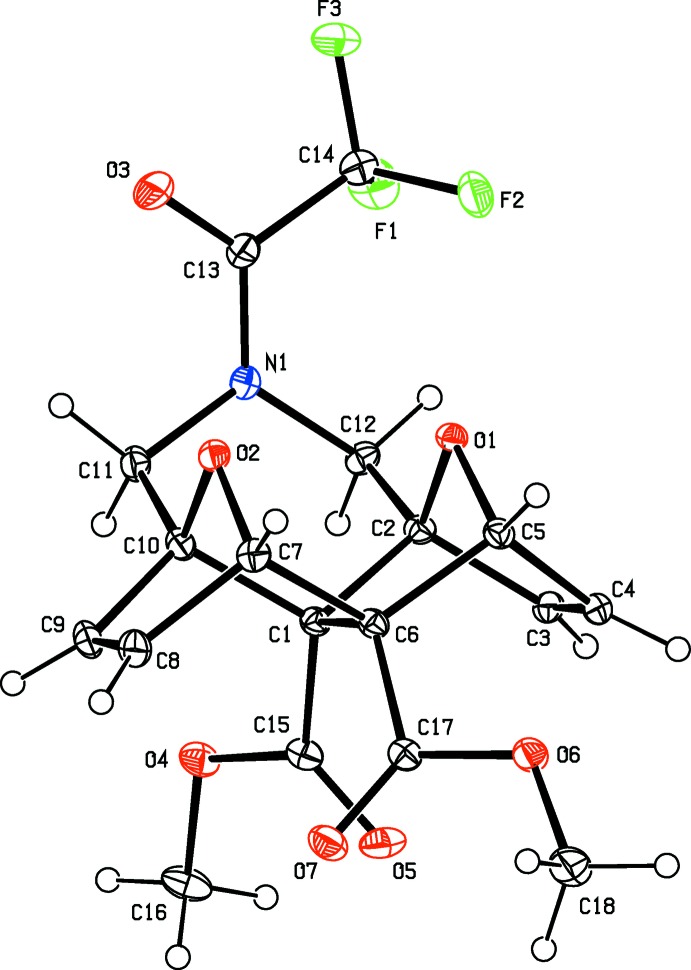
The mol­ecular structure of the title compound. Displacement ellipsoids are drawn at the 30% probability level. Hydrogen atoms are shown as spheres of arbitrary radius.

The mol­ecule of the title compound (Fig. 1 ▸) is made up from a fused cyclic system containing four five-membered rings (two di­hydro­furan and two tetra­hydro­furan) in the usual envelope conformations and a six-membered piperidine ring in a chair conformation. The latter is distorted because the environment of the N1 atom is inter­mediate between trigonal–planar and trigonal–pyramidal. The puckering parameters of the five-membered di­hydro­furan [*A* (O1/C1/C2/C5/C6), *B* (O2/C1/C6/C7/C10)] and tetra­hydro­furan [*C* (O1/C2–C5), *D* (O2/C7–C10)] rings are *A*: *Q*(2) = 0.5780 (15) Å, φ(2) = 359.75 (17)°; *B*: *Q*(2) = 0.5737 (16) Å, φ(2) = 4.53 (17)°; *C*: *Q*(2) = 0.5173 (15) Å, φ(2) = 179.60 (19)°; *D*: *Q*(2) = 0.5154 (16) Å, φ(2) = 178.2 (2)°. The puckering parameters of the six-membered piperidine ring (N1/C1/C2/C10–C12) are *Q*_T_ = 0.5312 (17) Å, θ = 9.58 (18)°, φ = 329.1 (11)°.

The mol­ecular conformations are stabilized by weak intra­molecular C—H⋯O and C—H⋯F inter­actions (Table 1 ▸) between methyl­ene groups (C11; C12) and a meth­oxy group and the –CF_3_ group, respectively. A rather unusual intra­molecular O**⋯**F inter­action between one of the oxygen bridgehead atoms (O1) and one of the F atoms of the –CF_3_ group [C5—O1⋯F2 = 2.9336 (16) Å; C5—O1⋯F2 = 153.60 (9)°] might help to consolidate the conformational arrangement.

## Supra­molecular features   

**Figure 2:**
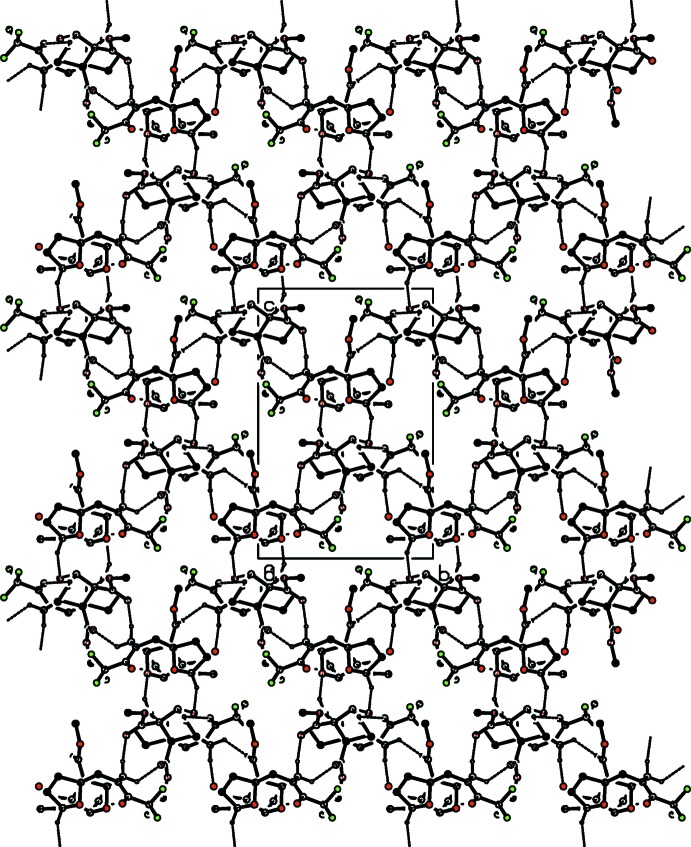
The crystal structure of the title compound in a view along [100], emphasizing the inter­molecular C—H⋯O and C—H⋯F hydrogen bonds (dashed lines).

Inter­molecular C—H⋯O inter­actions involving the O atoms of carbonyl groups, the oxygen bridgehead atoms and meth­oxy O atoms, as well as C—H⋯F hydrogen bonds define the crystal packing, which is shown in Fig. 2 ▸. These packing features lead to the formation of a three-dimensional network structure. C—H⋯π and π–π inter­actions are not observed, but H⋯H inter­actions dominate in the packing as detailed in the next section.

## Hirshfeld surface analysis   

**Figure 3:**
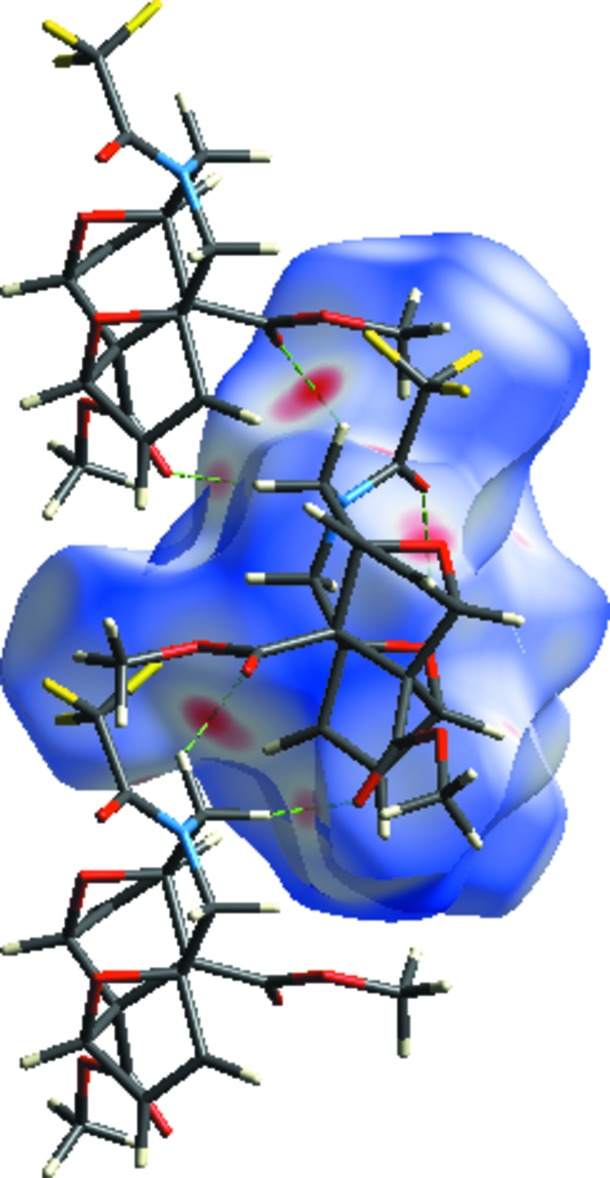
Hirshfeld surface of the title compound mapped over *d*_norm_.

**Figure 4:**
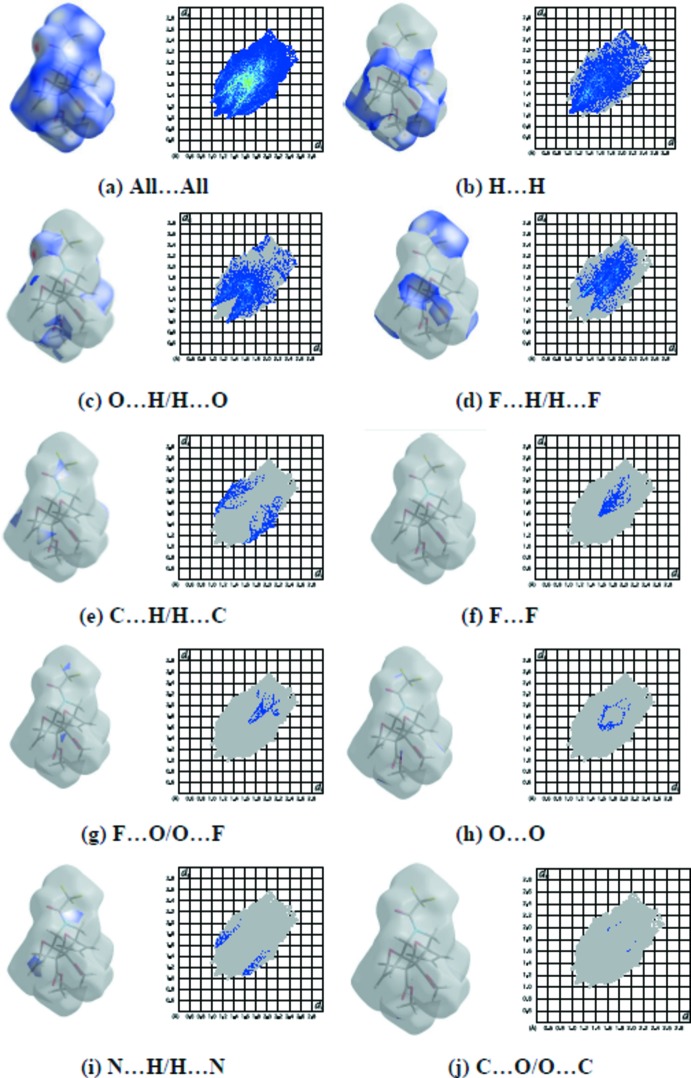
The two-dimensional fingerprint plots of the title compound, showing (*a*) all inter­actions, and delineated into (*b*) H⋯H, (*c*) O⋯H/ H⋯O, (*d*) F⋯H/H⋯F, (*e*) C⋯H/H⋯C, (*f*) F⋯F, (*g*) F⋯O/O⋯F, (*h*) O⋯O, (*i*) N⋯H/H⋯N and (*j*) C⋯O/O⋯C inter­actions [*d*_e_ and *d*_i_ represent the distances from a point on the Hirshfeld surface to the nearest atoms outside (external) and inside (inter­nal) the surface, respectively].

Hirshfeld surface and fingerprint plots were generated using *CrystalExplorer* (McKinnon *et al.*, 2007 ▸). Hirshfeld surfaces enable the visualization of inter­molecular inter­actions by different colors and color intensity, representing short or long contacts and indicating the relative strength of the inter­actions. Fig. 3 ▸ shows the Hirshfeld surface of the title compound mapped over *d*_norm_ where it is evident from the bright-red spots appearing near the oxygen atoms that these atoms play a significant role in the mol­ecular packing. The red spots represent closer contacts and negative *d*_norm_ values on the surface, corresponding to the C—H⋯O inter­actions. The percentage contributions of various contacts to the total Hirshfeld surface are given in Table 2 ▸ and are also shown as two-dimensional fingerprint plots in Fig. 4 ▸. The H⋯H inter­actions appear in the middle of the scattered points in the two-dimensional fingerprint plots with an overall contribution to the Hirshfeld surface of 35.6% (Fig. 4 ▸ *b*). The contribution from the O⋯H/H⋯O contacts, corresponding to C—H⋯O inter­actions, is represented by a pair of sharp spikes characteristic of a strong hydrogen-bonding inter­action (28.5%; Fig. 4 ▸ *c*). The contribution of the F⋯H/H⋯F inter­molecular contacts to the Hirshfeld surfaces is 23.8% (Fig. 4 ▸ *d*). The small percentage contributions from the remaining inter­atomic contacts are summarized in Table 2 ▸ and indicated by their fingerprint plots for C⋯H/H⋯C (Fig. 4 ▸ *e*), F⋯F (Fig. 4 ▸ *f*), F⋯O/O⋯F (Fig. 4 ▸ *g*), O⋯O (Fig. 4 ▸ *h*), N⋯H/H⋯N (Fig. 4 ▸ *i*) and C⋯O/O⋯C (Fig. 4 ▸ *j*). The large number of H⋯H, O⋯H/H⋯O and F⋯H/H⋯F inter­actions suggest that van der Waals inter­actions and hydrogen bonding play the major roles in the crystal packing (Hathwar *et al.*, 2015 ▸).

## Database survey   

A search of the Cambridge Structural Database (Version 5.39; Groom *et al.*, 2016 ▸) for similar structures showed the two closest are those of 2-benzyl-6a,9b-bis­(tri­fluoro­meth­yl)-2,3,6a,9b-tetra­hydro-1*H*,6*H*,7*H*-3a,6:7,9a-di­epoxy­benzo[*de*]iso­quinoline (CSD refcode HENLAQ; Borisova *et al.*, 2018*c* ▸) and 2-benzyl-4,5-bis­(tri­fluoro­meth­yl)-2,3,6a,9b-tetra­hydro-1*H*,6*H*,7*H*-3a,6:7,9a-di­epoxy­benzo[*de*]iso­quinoline (HEN­LEU; Borisova *et al.*, 2018*d* ▸). In the crystal of HENLAQ, inversion-related pairs of mol­ecules are linked into dimers by C—H⋯O hydrogen bonds. These dimers form sheets lying parallel to (100). C—H⋯π inter­actions are also observed in the crystal structure of HENLAQ, together with intra­molecular F⋯F contacts. The asymmetric unit of HENLEU contains two mol­ecules. In the crystal, mol­ecules are linked by C—H⋯O and C—H⋯F hydrogen bonds, forming columns along [010]. Likewise, C—H⋯π inter­actions and F⋯F intra­molecular contacts are also present.

## Synthesis and crystallization   

The synthesis of the title compound and its characterization by ^1^H NMR, ^13^C NMR, IR and HRMS spectroscopy have previously been reported (Borisova *et al.*, 2018*a* ▸). Dimethyl acetyl­enedi­carboxyl­ate (DMAD, 1.84 ml, 0.015 mol) was added to a solution of 2,2,2-tri­fluoro-*N*,*N*-bis­(furan-2-yl­meth­yl)acetamide (0.01 mol) in benzene (30 ml). The mixture was heated at reflux for 15.5–40 h at 353 K (GC–MS monitoring until disappearance of the starting material). The reaction mixture was cooled and left overnight at room temperature. The solvent was removed under reduced pressure. The residue (brown oil) was triturated with diethyl ether. The obtained crystals were filtered off and recrystallized from hexa­ne/EtOAc (*v*:*v* = 2:1) to give the pure compound as a white powder (2.57 g, 6.2 mmol, yield 62%). *R*_f_ = 0.56 (EtOAc/hexane, 2:1, Sorbfil). M.p. 467.2–467.9 K (from hexa­ne/EtOAc). ^1^H NMR (400 MHz, CDCl_3_): δ 6.74–6.71 (2H, *m*, H-4 and H-9), 6.46 (2H, *dd*, *J* = 2.3 and *J* = 5.5 Hz, H-5 and H-8), 5.14 (2H, *br s*, H-6 and H-7), 5.10 (1H, *d*, *J* = 14.9 Hz, H-1A), 4.43 (1H, *br d*, *J* = 14.9 Hz, H-3A), 4.08 (1H, *d*, *J* = 14.9 Hz, H-3B), 3.64 (6H, *s*, 2 × CO_2_Me), 3.59 (1H, *d*, *J* = 14.9, H-1B). ^13^C NMR (100 MHz, CDCl_3_): δ 170.1 (2 × CO_2_Me), 157.2 (*q*, *J* = 35.5 Hz, F3C—C), 141.2 (C-5 and C-8), 137.5 (C-4 and C-9), 116.4 (*q*, *J* = 288.1 Hz, CF_3_), 87.1 (C-3a and C-9a), 83.8 (C-6 and C-7), 71.4 and 68.8 (C-9 and C-6a), 52.4 (2 × CO_2_Me), 44.8 (*q*, *J* = 3.8 Hz, C-1), 42.4 (C-3). ^19^F NMR (282 MHz, CDCl_3_): δ −67.7 (*s*, CF_3_). IR ν_max_/cm^−1^ (KBr): 3109, 3055, 2956, 1713, 1688, 1197. HRMS (ESI–TOF): calculated for C_18_H_16_F_3_NO_7_ [*M* + H]^+^, 415.0879; found, 415.0889.

## Refinement details   

Crystal data, data collection and structure refinement details are summarized in Table 3 ▸. All H atoms were fixed and allowed to ride on the parent atoms, with C—H = 0.95–1.00 Å, and with *U*_iso_(H) = 1.5*U*_eq_(C) for methyl H atoms and 1.2*U*_eq_(C) for all other H atoms. Eight outliers [(101), (011), (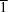
01), (002), (110), (363), (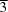
03), (111)] were omitted in the final cycles of refinement.

## Supplementary Material

Crystal structure: contains datablock(s) I, global. DOI: 10.1107/S2056989018014305/wm5463sup1.cif

Structure factors: contains datablock(s) I. DOI: 10.1107/S2056989018014305/wm5463Isup2.hkl

CCDC reference: 1872524

Additional supporting information:  crystallographic information; 3D view; checkCIF report

## Figures and Tables

**Table 1:** Hydrogen-bond geometry (Å, °)

*D*—H⋯*A*	*D*—H	H⋯*A*	*D*⋯*A*	*D*—H⋯*A*
C4—H4⋯O3^i^	0.95	2.44	3.116 (2)	128
C5—H5⋯O2^ii^	1.00	2.60	3.1960 (19)	118
C7—H7⋯O1^ii^	1.00	2.54	3.2091 (19)	124
C11—H11*B*⋯O4	0.99	2.57	3.093 (2)	113
C12—H12*A*⋯O7^iii^	0.99	2.52	3.328 (2)	138
C12—H12*B*⋯O5^iii^	0.99	2.34	3.030 (2)	127
C12—H12*B*⋯F1	0.99	2.40	3.043 (2)	122
C12—H12*B*⋯F2	0.99	2.33	2.962 (2)	121
C16—H16*A*⋯F3^iv^	0.98	2.62	3.475 (2)	146

Symmetry codes: (i) 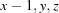; (ii) 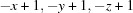; (iii) 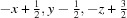; (iv) 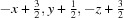.

**Table 2:** Percentage contributions of inter­atomic contacts to the Hirshfeld surface for the title compound

Contact	Percentage contribution
H⋯H	35.6
O⋯H/H⋯O	28.5
F⋯H/H⋯F	23.8
C⋯H/H⋯C	5.5
F⋯F	2.7
F⋯O/O⋯F	1.6
N⋯H/H⋯N	1.1
O⋯O	1.1
C⋯O/O⋯C	0.2

**Table 3:** Experimental details

Crystal data	
Chemical formula	C_18_H_16_F_3_NO_7_
*M*_r_	415.32
Crystal system, space group	Monoclinic, *P*2_1_/*n*
Temperature (K)	150
*a*, *b*, *c* (Å)	8.7661 (2), 11.2908 (3), 17.5089 (4)
β (°)	96.021 (1)
*V* (Å^3^)	1723.41 (7)
*Z*	4
Radiation type	Mo *K*α
μ (mm^−1^)	0.14
Crystal size (mm)	0.35 × 0.32 × 0.30

Data collection	
Diffractometer	Bruker APEXII CCD
Absorption correction	Multi-scan (*SADABS*; Krause *et al.*, 2015 ▸)
*T*_min_, *T*_max_	0.942, 0.946
No. of measured, independent and observed [*I* > 2σ(*I*)] reflections	11170, 3496, 2739
*R*_int_	0.028
(sin θ/λ)_max_ (Å^−1^)	0.626

Refinement	
*R*[*F* ^2^ > 2σ(*F* ^2^)], *wR*(*F* ^2^), *S*	0.036, 0.091, 1.01
No. of reflections	3496
No. of parameters	264
H-atom treatment	H-atom parameters constrained
Δρ_max_, Δρ_min_ (e Å^−3^)	0.30, −0.25

Computer programs: *APEX2* and *SAINT* (Bruker, 2007 ▸), *SHELXS97* (Sheldrick, 2008 ▸), *SHELXL2014* (Sheldrick, 2015 ▸), *ORTEP-3 for Windows* (Farrugia, 2012 ▸) and *PLATON* (Spek, 2009 ▸).

## supplementary crystallographic information

### Crystal data

**Table:**

C_18_H_16_F_3_NO_7_	*F*(000) = 856
*M**_r_* = 415.32	*D*_x_ = 1.601 Mg m^−^^3^
Monoclinic, *P*2_1_/*n*	Mo *K*α radiation, λ = 0.71073 Å
*a* = 8.7661 (2) Å	Cell parameters from 3572 reflections
*b* = 11.2908 (3) Å	θ = 3.0–25.9°
*c* = 17.5089 (4) Å	µ = 0.14 mm^−^^1^
β = 96.021 (1)°	*T* = 150 K
*V* = 1723.41 (7) Å^3^	Block, colourless
*Z* = 4	0.35 × 0.32 × 0.30 mm

### Data collection

**Table:**

Bruker APEXII CCD diffractometer	2739 reflections with *I* > 2σ(*I*)
φ and ω scans	*R*_int_ = 0.028
Absorption correction: multi-scan (*SADABS*; Krause *et al.*, 2015)	θ_max_ = 26.4°, θ_min_ = 3.1°
*T*_min_ = 0.942, *T*_max_ = 0.946	*h* = −10→10
11170 measured reflections	*k* = −14→11
3496 independent reflections	*l* = −21→20

### Refinement

**Table:**

Refinement on *F*^2^	0 restraints
Least-squares matrix: full	Hydrogen site location: inferred from neighbouring sites
*R*[*F*^2^ > 2σ(*F*^2^)] = 0.036	H-atom parameters constrained
*wR*(*F*^2^) = 0.091	*w* = 1/[σ^2^(*F*_o_^2^) + (0.0391*P*)^2^ + 0.8462*P*] where *P* = (*F*_o_^2^ + 2*F*_c_^2^)/3
*S* = 1.01	(Δ/σ)_max_ < 0.001
3496 reflections	Δρ_max_ = 0.30 e Å^−^^3^
264 parameters	Δρ_min_ = −0.25 e Å^−^^3^

### Special details

**Table:**

Geometry. All esds (except the esd in the dihedral angle between two l.s. planes) are estimated using the full covariance matrix. The cell esds are taken into account individually in the estimation of esds in distances, angles and torsion angles; correlations between esds in cell parameters are only used when they are defined by crystal symmetry. An approximate (isotropic) treatment of cell esds is used for estimating esds involving l.s. planes.

### Fractional atomic coordinates and isotropic or equivalent isotropic displacement parameters (Å^2^)

**Table:**

	*x*	*y*	*z*	*U*_iso_*/*U*_eq_	
C1	0.39473 (17)	0.49611 (14)	0.67777 (9)	0.0151 (3)	
C2	0.34636 (17)	0.36454 (14)	0.65512 (9)	0.0159 (3)	
C3	0.17517 (18)	0.35392 (15)	0.66061 (10)	0.0188 (4)	
H3	0.125910	0.313775	0.698918	0.023*	
C4	0.10926 (18)	0.41275 (15)	0.60044 (9)	0.0190 (4)	
H4	0.002414	0.423058	0.586488	0.023*	
C5	0.23948 (17)	0.46040 (14)	0.55824 (9)	0.0166 (3)	
H5	0.211134	0.478143	0.502604	0.020*	
C6	0.31682 (17)	0.56580 (14)	0.60653 (9)	0.0156 (3)	
C7	0.46580 (18)	0.61837 (14)	0.57545 (9)	0.0181 (3)	
H7	0.450625	0.648514	0.521478	0.022*	
C8	0.53785 (19)	0.70697 (16)	0.63430 (10)	0.0223 (4)	
H8	0.534938	0.790901	0.630565	0.027*	
C9	0.60537 (19)	0.64310 (16)	0.69172 (10)	0.0215 (4)	
H9	0.663054	0.671359	0.737022	0.026*	
C10	0.57077 (18)	0.51491 (15)	0.66959 (9)	0.0173 (3)	
C11	0.67413 (18)	0.41488 (15)	0.70014 (9)	0.0196 (4)	
H11A	0.777907	0.426465	0.683886	0.023*	
H11B	0.683028	0.415179	0.756980	0.023*	
C12	0.45739 (18)	0.27365 (15)	0.69121 (9)	0.0187 (3)	
H12A	0.456518	0.274715	0.747704	0.022*	
H12B	0.426248	0.193647	0.672394	0.022*	
C13	0.68211 (18)	0.24366 (16)	0.61696 (10)	0.0213 (4)	
C14	0.61562 (19)	0.12361 (17)	0.58786 (11)	0.0268 (4)	
C15	0.34324 (19)	0.52840 (15)	0.75488 (9)	0.0187 (4)	
C16	0.4132 (2)	0.54441 (18)	0.88782 (9)	0.0294 (4)	
H16A	0.502378	0.535376	0.926090	0.044*	
H16B	0.332265	0.489567	0.899623	0.044*	
H16C	0.375106	0.625921	0.888922	0.044*	
C17	0.20610 (18)	0.66654 (15)	0.61749 (9)	0.0171 (3)	
C18	−0.0373 (2)	0.74427 (17)	0.57152 (12)	0.0332 (5)	
H18A	−0.136339	0.716249	0.547019	0.050*	
H18B	−0.001422	0.810491	0.541928	0.050*	
H18C	−0.048909	0.770680	0.623910	0.050*	
N1	0.61226 (15)	0.30034 (12)	0.67137 (8)	0.0174 (3)	
O1	0.35051 (12)	0.36756 (10)	0.57348 (6)	0.0157 (2)	
O2	0.57083 (12)	0.52159 (10)	0.58756 (6)	0.0171 (3)	
O3	0.79898 (15)	0.27623 (13)	0.59189 (8)	0.0386 (4)	
O4	0.45760 (13)	0.51821 (11)	0.81186 (6)	0.0234 (3)	
O5	0.21495 (14)	0.55547 (12)	0.76498 (7)	0.0274 (3)	
O6	0.07292 (13)	0.64922 (10)	0.57403 (7)	0.0226 (3)	
O7	0.23490 (14)	0.75410 (11)	0.65517 (7)	0.0263 (3)	
F1	0.60895 (14)	0.04589 (10)	0.64497 (7)	0.0422 (3)	
F2	0.47566 (12)	0.13074 (10)	0.55061 (6)	0.0350 (3)	
F3	0.70476 (14)	0.07621 (12)	0.53923 (8)	0.0509 (4)	

### Atomic displacement parameters (Å^2^)

**Table:**

	*U*^11^	*U*^22^	*U*^33^	*U*^12^	*U*^13^	*U*^23^
C1	0.0169 (8)	0.0155 (8)	0.0130 (8)	−0.0009 (6)	0.0023 (6)	−0.0006 (6)
C2	0.0181 (8)	0.0163 (8)	0.0139 (8)	−0.0018 (6)	0.0051 (6)	−0.0002 (6)
C3	0.0172 (8)	0.0149 (8)	0.0254 (9)	−0.0042 (6)	0.0082 (7)	−0.0020 (7)
C4	0.0163 (8)	0.0167 (9)	0.0244 (9)	−0.0025 (6)	0.0033 (6)	−0.0048 (7)
C5	0.0173 (8)	0.0169 (8)	0.0153 (8)	0.0008 (6)	0.0002 (6)	−0.0011 (7)
C6	0.0185 (8)	0.0150 (8)	0.0134 (8)	−0.0012 (6)	0.0022 (6)	−0.0005 (6)
C7	0.0199 (8)	0.0168 (9)	0.0179 (8)	−0.0009 (6)	0.0039 (6)	0.0027 (7)
C8	0.0226 (9)	0.0184 (9)	0.0268 (9)	−0.0068 (7)	0.0060 (7)	−0.0007 (7)
C9	0.0213 (8)	0.0222 (9)	0.0205 (9)	−0.0071 (7)	−0.0002 (7)	−0.0038 (7)
C10	0.0191 (8)	0.0184 (9)	0.0144 (8)	−0.0048 (6)	0.0024 (6)	−0.0019 (7)
C11	0.0173 (8)	0.0230 (9)	0.0180 (8)	−0.0029 (7)	−0.0003 (6)	−0.0014 (7)
C12	0.0181 (8)	0.0198 (9)	0.0193 (8)	−0.0011 (7)	0.0064 (6)	0.0027 (7)
C13	0.0171 (8)	0.0241 (10)	0.0225 (9)	0.0007 (7)	0.0020 (7)	−0.0008 (7)
C14	0.0226 (9)	0.0268 (10)	0.0315 (10)	0.0019 (7)	0.0048 (7)	−0.0048 (8)
C15	0.0254 (9)	0.0149 (8)	0.0161 (8)	−0.0005 (7)	0.0042 (7)	0.0006 (7)
C16	0.0470 (11)	0.0304 (11)	0.0114 (8)	0.0037 (9)	0.0057 (8)	−0.0025 (8)
C17	0.0211 (8)	0.0172 (9)	0.0131 (8)	−0.0012 (6)	0.0030 (6)	0.0016 (7)
C18	0.0299 (10)	0.0263 (11)	0.0409 (11)	0.0115 (8)	−0.0074 (8)	−0.0080 (9)
N1	0.0158 (7)	0.0180 (7)	0.0184 (7)	−0.0010 (5)	0.0016 (5)	0.0014 (6)
O1	0.0176 (5)	0.0157 (6)	0.0141 (6)	−0.0001 (4)	0.0034 (4)	−0.0016 (4)
O2	0.0170 (6)	0.0201 (6)	0.0148 (6)	−0.0021 (5)	0.0039 (4)	0.0005 (5)
O3	0.0259 (7)	0.0424 (9)	0.0509 (9)	−0.0105 (6)	0.0205 (6)	−0.0156 (7)
O4	0.0297 (7)	0.0287 (7)	0.0116 (6)	0.0004 (5)	0.0019 (5)	−0.0020 (5)
O5	0.0296 (7)	0.0322 (8)	0.0215 (6)	0.0095 (6)	0.0086 (5)	−0.0015 (5)
O6	0.0212 (6)	0.0194 (6)	0.0261 (6)	0.0044 (5)	−0.0023 (5)	−0.0039 (5)
O7	0.0296 (7)	0.0210 (7)	0.0272 (7)	0.0035 (5)	−0.0020 (5)	−0.0069 (5)
F1	0.0515 (7)	0.0218 (6)	0.0523 (8)	0.0029 (5)	0.0002 (6)	0.0056 (5)
F2	0.0279 (6)	0.0320 (6)	0.0427 (7)	−0.0024 (5)	−0.0073 (5)	−0.0102 (5)
F3	0.0405 (7)	0.0496 (8)	0.0662 (9)	−0.0048 (6)	0.0225 (6)	−0.0341 (7)

### Geometric parameters (Å, º)

**Table:**

C1—C15	1.512 (2)	C11—N1	1.471 (2)
C1—C6	1.569 (2)	C11—H11A	0.9900
C1—C10	1.579 (2)	C11—H11B	0.9900
C1—C2	1.584 (2)	C12—N1	1.467 (2)
C2—O1	1.4341 (19)	C12—H12A	0.9900
C2—C12	1.507 (2)	C12—H12B	0.9900
C2—C3	1.519 (2)	C13—O3	1.213 (2)
C3—C4	1.326 (2)	C13—N1	1.347 (2)
C3—H3	0.9500	C13—C14	1.541 (3)
C4—C5	1.522 (2)	C14—F3	1.327 (2)
C4—H4	0.9500	C14—F2	1.330 (2)
C5—O1	1.4363 (19)	C14—F1	1.336 (2)
C5—C6	1.572 (2)	C15—O5	1.196 (2)
C5—H5	1.0000	C15—O4	1.343 (2)
C6—C17	1.520 (2)	C16—O4	1.455 (2)
C6—C7	1.582 (2)	C16—H16A	0.9800
C7—O2	1.4303 (19)	C16—H16B	0.9800
C7—C8	1.525 (2)	C16—H16C	0.9800
C7—H7	1.0000	C17—O7	1.2006 (19)
C8—C9	1.325 (2)	C17—O6	1.3393 (19)
C8—H8	0.9500	C18—O6	1.441 (2)
C9—C10	1.521 (2)	C18—H18A	0.9800
C9—H9	0.9500	C18—H18B	0.9800
C10—O2	1.4382 (19)	C18—H18C	0.9800
C10—C11	1.510 (2)		
			
C15—C1—C6	116.28 (13)	C9—C10—C1	106.00 (13)
C15—C1—C10	115.76 (13)	N1—C11—C10	110.50 (12)
C6—C1—C10	102.03 (12)	N1—C11—H11A	109.5
C15—C1—C2	110.64 (13)	C10—C11—H11A	109.5
C6—C1—C2	100.86 (12)	N1—C11—H11B	109.5
C10—C1—C2	109.99 (12)	C10—C11—H11B	109.5
O1—C2—C12	110.57 (12)	H11A—C11—H11B	108.1
O1—C2—C3	101.16 (12)	N1—C12—C2	109.50 (13)
C12—C2—C3	121.25 (13)	N1—C12—H12A	109.8
O1—C2—C1	101.14 (11)	C2—C12—H12A	109.8
C12—C2—C1	112.92 (13)	N1—C12—H12B	109.8
C3—C2—C1	107.37 (13)	C2—C12—H12B	109.8
C4—C3—C2	105.15 (14)	H12A—C12—H12B	108.2
C4—C3—H3	127.4	O3—C13—N1	125.15 (16)
C2—C3—H3	127.4	O3—C13—C14	116.83 (15)
C3—C4—C5	106.05 (14)	N1—C13—C14	117.88 (14)
C3—C4—H4	127.0	F3—C14—F2	106.54 (15)
C5—C4—H4	127.0	F3—C14—F1	106.87 (16)
O1—C5—C4	100.37 (12)	F2—C14—F1	107.23 (14)
O1—C5—C6	101.94 (12)	F3—C14—C13	109.82 (14)
C4—C5—C6	108.05 (13)	F2—C14—C13	113.97 (15)
O1—C5—H5	114.9	F1—C14—C13	112.02 (15)
C4—C5—H5	114.9	O5—C15—O4	123.51 (15)
C6—C5—H5	114.9	O5—C15—C1	124.53 (15)
C17—C6—C1	120.34 (13)	O4—C15—C1	111.90 (14)
C17—C6—C5	112.90 (13)	O4—C16—H16A	109.5
C1—C6—C5	100.09 (12)	O4—C16—H16B	109.5
C17—C6—C7	108.88 (13)	H16A—C16—H16B	109.5
C1—C6—C7	98.94 (12)	O4—C16—H16C	109.5
C5—C6—C7	115.09 (13)	H16A—C16—H16C	109.5
O2—C7—C8	100.74 (12)	H16B—C16—H16C	109.5
O2—C7—C6	101.77 (12)	O7—C17—O6	123.57 (15)
C8—C7—C6	108.18 (13)	O7—C17—C6	125.81 (15)
O2—C7—H7	114.8	O6—C17—C6	110.43 (13)
C8—C7—H7	114.8	O6—C18—H18A	109.5
C6—C7—H7	114.8	O6—C18—H18B	109.5
C9—C8—C7	106.02 (15)	H18A—C18—H18B	109.5
C9—C8—H8	127.0	O6—C18—H18C	109.5
C7—C8—H8	127.0	H18A—C18—H18C	109.5
C8—C9—C10	105.27 (14)	H18B—C18—H18C	109.5
C8—C9—H9	127.4	C13—N1—C12	124.77 (14)
C10—C9—H9	127.4	C13—N1—C11	118.78 (13)
O2—C10—C11	109.26 (13)	C12—N1—C11	114.62 (13)
O2—C10—C9	100.58 (13)	C2—O1—C5	96.60 (11)
C11—C10—C9	121.69 (14)	C7—O2—C10	96.93 (11)
O2—C10—C1	101.56 (11)	C15—O4—C16	114.29 (13)
C11—C10—C1	115.03 (13)	C17—O6—C18	116.74 (13)
			
C15—C1—C2—O1	158.98 (12)	C6—C1—C10—C9	73.08 (14)
C6—C1—C2—O1	35.33 (13)	C2—C1—C10—C9	179.49 (12)
C10—C1—C2—O1	−71.87 (14)	O2—C10—C11—N1	−65.07 (16)
C15—C1—C2—C12	−82.85 (16)	C9—C10—C11—N1	178.56 (14)
C6—C1—C2—C12	153.50 (12)	C1—C10—C11—N1	48.35 (17)
C10—C1—C2—C12	46.30 (16)	O1—C2—C12—N1	57.13 (17)
C15—C1—C2—C3	53.42 (16)	C3—C2—C12—N1	175.15 (14)
C6—C1—C2—C3	−70.24 (14)	C1—C2—C12—N1	−55.37 (17)
C10—C1—C2—C3	−177.43 (13)	O3—C13—C14—F3	0.8 (2)
O1—C2—C3—C4	−32.33 (16)	N1—C13—C14—F3	−175.17 (15)
C12—C2—C3—C4	−154.93 (15)	O3—C13—C14—F2	−118.68 (18)
C1—C2—C3—C4	73.22 (16)	N1—C13—C14—F2	65.4 (2)
C2—C3—C4—C5	−0.44 (17)	O3—C13—C14—F1	119.35 (18)
C3—C4—C5—O1	32.93 (16)	N1—C13—C14—F1	−56.6 (2)
C3—C4—C5—C6	−73.38 (16)	C6—C1—C15—O5	35.8 (2)
C15—C1—C6—C17	4.8 (2)	C10—C1—C15—O5	155.57 (16)
C10—C1—C6—C17	−122.10 (14)	C2—C1—C15—O5	−78.4 (2)
C2—C1—C6—C17	124.52 (14)	C6—C1—C15—O4	−146.90 (14)
C15—C1—C6—C5	−119.37 (14)	C10—C1—C15—O4	−27.14 (19)
C10—C1—C6—C5	113.70 (12)	C2—C1—C15—O4	98.85 (15)
C2—C1—C6—C5	0.31 (13)	C1—C6—C17—O7	58.2 (2)
C15—C1—C6—C7	122.99 (14)	C5—C6—C17—O7	176.13 (15)
C10—C1—C6—C7	−3.95 (14)	C7—C6—C17—O7	−54.7 (2)
C2—C1—C6—C7	−117.33 (12)	C1—C6—C17—O6	−126.65 (15)
O1—C5—C6—C17	−165.16 (12)	C5—C6—C17—O6	−8.77 (18)
C4—C5—C6—C17	−59.95 (17)	C7—C6—C17—O6	120.36 (14)
O1—C5—C6—C1	−35.94 (13)	O3—C13—N1—C12	167.96 (17)
C4—C5—C6—C1	69.27 (14)	C14—C13—N1—C12	−16.5 (2)
O1—C5—C6—C7	68.99 (15)	O3—C13—N1—C11	4.3 (3)
C4—C5—C6—C7	174.20 (13)	C14—C13—N1—C11	179.81 (14)
C17—C6—C7—O2	165.38 (12)	C2—C12—N1—C13	−101.78 (18)
C1—C6—C7—O2	38.92 (14)	C2—C12—N1—C11	62.52 (17)
C5—C6—C7—O2	−66.71 (16)	C10—C11—N1—C13	106.74 (16)
C17—C6—C7—C8	59.79 (16)	C10—C11—N1—C12	−58.57 (17)
C1—C6—C7—C8	−66.68 (15)	C12—C2—O1—C5	−178.88 (12)
C5—C6—C7—C8	−172.31 (13)	C3—C2—O1—C5	51.41 (13)
O2—C7—C8—C9	−31.04 (16)	C1—C2—O1—C5	−59.02 (12)
C6—C7—C8—C9	75.27 (16)	C4—C5—O1—C2	−51.23 (13)
C7—C8—C9—C10	−1.76 (17)	C6—C5—O1—C2	59.90 (13)
C8—C9—C10—O2	33.85 (16)	C8—C7—O2—C10	50.57 (13)
C8—C9—C10—C11	154.49 (15)	C6—C7—O2—C10	−60.77 (13)
C8—C9—C10—C1	−71.55 (16)	C11—C10—O2—C7	179.05 (12)
C15—C1—C10—O2	−158.88 (13)	C9—C10—O2—C7	−51.80 (13)
C6—C1—C10—O2	−31.61 (14)	C1—C10—O2—C7	57.12 (13)
C2—C1—C10—O2	74.81 (14)	O5—C15—O4—C16	−0.7 (2)
C15—C1—C10—C11	83.27 (17)	C1—C15—O4—C16	−178.05 (14)
C6—C1—C10—C11	−149.45 (13)	O7—C17—O6—C18	2.2 (2)
C2—C1—C10—C11	−43.04 (17)	C6—C17—O6—C18	−173.02 (14)
C15—C1—C10—C9	−54.19 (17)		

### Hydrogen-bond geometry (Å, º)

**Table:**

*D*—H···*A*	*D*—H	H···*A*	*D*···*A*	*D*—H···*A*
C4—H4···O3^i^	0.95	2.44	3.116 (2)	128
C5—H5···O2^ii^	1.00	2.60	3.1960 (19)	118
C7—H7···O1^ii^	1.00	2.54	3.2091 (19)	124
C11—H11*B*···O4	0.99	2.57	3.093 (2)	113
C12—H12*A*···O7^iii^	0.99	2.52	3.328 (2)	138
C12—H12*B*···O5^iii^	0.99	2.34	3.030 (2)	127
C12—H12*B*···F1	0.99	2.40	3.043 (2)	122
C12—H12*B*···F2	0.99	2.33	2.962 (2)	121
C16—H16*A*···F3^iv^	0.98	2.62	3.475 (2)	146

Symmetry codes: (i) *x*−1, *y*, *z*; (ii) −*x*+1, −*y*+1, −*z*+1; (iii) −*x*+1/2, *y*−1/2, −*z*+3/2; (iv) −*x*+3/2, *y*+1/2, −*z*+3/2.
